# Commentary: A view from down under

**DOI:** 10.1155/S1463924680000016

**Published:** 1980


### Commentary

### A view from down under

After visits over the years to countries east of Suez, a lengthy sojourn in the capital cities of Australia affords an opportunity to review the situation with regard to laboratory automation in the geographical area usually referred to as the Far East.

Many of the countries included are unique and together they represent a wide range of economic, social and industrial developments, therefore generalised comment is not possible except in-so-far as the major suppliers of automatic instruments are based mainly in the 'west', and the features common to distant markets are apparent.

Sales and maintenance are carried out to a much greater extent by agents, long delays can occur before new instruments and developments are introduced and since many are not economically viable because of the length of supply lines or the small size of markets, the range of instruments available is considerably reduced. It is evident however, that when a manufacturer makes a determined effort to introduce equipment he can achieve remarkable success if only because of the lack of determined competition in many areas. The market is far from being uncritical however; some of the best evaluations of automatic instruments for clinical chemistry have been completed, for example, in Adelaide.

In developing countries such as Australia, the population to be served for health care might be comparatively small (14 million) but quality and service is high. The national economy is buoyant and in the recent past there has been considerable investment in the hospital sector. Some of the world's newest and best equipped hospitals are to be found in the major cities.

At the opposite end of the population scale, China, with approximately one quarter of the world's population (some 900 million) presents a somewhat different picture. Until recently there was little scientific contact outside the country, but the progress of science has been impressive. Chairman Mao's policy of not trailing behind the rest of the world but developing independently, together with equal care and opportunity for all has meant that the import of 'Western' technology has been minimal, the need for automation not being great because of the plentiful supply of labour and absence of centralisation. The overall picture in China, therefore, is of a wide distribution for instance of health care, with a very large number of relatively simple manual colorimeters and few centres in the large cities needing and using modest systems of automation. With the country's rapid economic advance and new outward look there is enormous development potential which in the more industrial areas of chemistry will probably be on conventional lines, but in the health sector, growth will almost certainly follow the already successful approach with a wide distribution of relatively small scale, broad-based facilities.

The usage and range of instruments in Japan differs little from those in other highly developed nations. A somewhat belated but now rapidly growing local interest in the design and production of automatic systems resulted in a number of Japanese made machines, some large and complex, being in use in the country but not as yet exported on any scale.

India also has a rapidly growing chemical instrument manufacturing industry, but it differs from the Japanese for two reasons. Firstly, in the absence of very large manufacturing firms interested and willing to enter the market, large specialist companies have had to develop, and since these do not have the advantage of already functioning efficient design engineering departments, the complexity of the machines which can be produced is restricted. Secondly, the requirements of the country are more for small, low cost, efficient, manually operated instruments rather than for large computer-controlled fast analysers. Since both China and India have similar requirements with matching and growing production, it is possible that by specialising in the type of instrument required by developing nations in general, they may find a useful outlet into Third World markets.

Nepal is an example of a small country which comparatively recently had no motor roads or vehicular access, let alone laboratory based medicine. Yet with WHO guidance and outside monetary aid, facilities are rapidly expanding outside Katmandu and like all other countries developing similarly, the requirement is for inexpensive, simply operated instruments which will function for many years without maintenance and independent of mains electricity supplies. Other countries in the area fall between the extremes mentioned, but as in the 'West' for those without advanced services and economies there are many instances where equipment designed for 'Western' civilisations has been installed at considerable expense, only to break down permanently at an early stage or at best, operate at a low level of efficiency. Occasionally however, there are to be found items of advanced technology such as mass spectrometers, operating at high efficiency under the care of dedicated and resourceful operators.

In summary, therefore, in the use of chemical automation, east of Suez things are different, but considering the very different conditions and the distances involved, the differences are not as great as might be expected.

*F.L. Mitchell*

### From the Editor's desk

As the *Journal of Automatic Chemistry* moves into its second volume I can report a steadily growing subscription list, a rapidly increasing interest from the commercial sector and a considerable input of papers for publication. Interest in the journal is now almost world wide and with the citation of many of our papers in other scientific journals the subscription should grow rapidly. Whilst there is often concern at the proliferation of new journals, the *Journal of Automatic Chemistry* attempts to be unique. It is multidisciplinary by the nature of its subject areas and also covers wider issues including education, management, and economics. Educational aspects have been widely covered in Volume I although no doubt there is still more to say. Management is the subject of Foreman's article in this issue and economic aspects are treated in some detail by Craig. The latter which was presented at clinical meetings at Brighton and Singapore, relates to a particular company's approach to costing, but is also of general applicability. Whilst these papers relate on the one hand to an industrial environment and the other to clinical laboratories each has value to readers in the other disciplines. Indeed it is the mechanism to promote the cross fertilisation of ideas and concepts between different disciplines that I see as one of the most important functions of the journal. Initially the concept was to foster the exchange of ideas between clinical

